# Recognition of Zinc Transporter 8 and MAP3865c Homologous Epitopes by Hashimoto's Thyroiditis Subjects from Sardinia: A Common Target with Type 1 Diabetes?

**DOI:** 10.1371/journal.pone.0097621

**Published:** 2014-05-15

**Authors:** Speranza Masala, Davide Cossu, Mario Palermo, Leonardo Antonio Sechi

**Affiliations:** 1 Università degli Studi di Sassari, Dipartimento di Scienze Biomediche, Sezione di Microbiologia e Virologia, Sassari, Italy; 2 Department of Endocrinology, Azienda Sanitaria Locale (ASL) 1, Sassari, Italy; University of Siena, Italy

## Abstract

*Mycobacterium avium* subspecies *paratuberculosis* (MAP) asymptomatic infection has been previously linked to Type 1 diabetes (T1D) and Multiple Sclerosis. An association between MAP infection and Hashimoto's thyroiditis (HT) was also proposed only in a case report. This study aimed to investigate the robustness of the latter association, testing a large cohort of HT and healthy control (HCs) subjects, all from Sardinia. Prevalence of anti-MAP3865c Abs was assessed by indirect enzyme-linked immunosorbent assay (ELISA). Moreover, given that human ZnT8 is specifically expressed in the pancreatic β-cells, in the follicle epithelial cells and in the parafollicular cells of the thyroid gland, we also tested ZnT8 epitopes homologues to the MAP3865c immunodominant peptides previously identified. Indeed, Abs targeting MAP3865c and ZnT8 homologous regions display similar frequencies in patients and controls, thus suggesting that Abs recognizing these epitopes could be cross-reactive. A statistically significant difference was found between HT patients and HCs when analyzing the humoral response mounted against MAP3865c/ZnT8 homologues epitopes. To our knowledge, this is the first report, which provides statistically significant evidence sustaining the existence of an association between MAP sero-reactivity and HT. Further studies are required to investigate the relevance of MAP to HT, aimed at deciphering if this pathogen can be at play in triggering this autoimmune disease. Likewise, genetic polymorphism of the host, and other environmental factors need to be investigated.

## Introduction

Hashimoto's thyroiditis (HT) is a chronic inflammatory condition affecting the thyroid gland. The hallmark of this disease is the abundant lymphocyte B and T infiltrate, resulting in thyroid destruction. It involves both humoral and cellular responses against two thyroid autoantigens: thyroid peroxidase (TPO) and thyroglobulin (TG) [1]. HT is a multifactorial disease stemming from an interaction between genetic and environmental risk factors [2], [3]. It has been proposed that *Mycobacterium avium* subspecies *paratuberculosis* (MAP) may be one of the environmental factor at play in triggering HT disease [4], [5], but the assumption made was not supported by statistically significant data.

MAP specifically colonizes the mucosa-associated lymphoid tissue (MALT) of the small intestine, where it resides inside the intraepithelial macrophages [6] causing Johne's disease (JD) in ruminants [7] and it is associated with Crohn's disease (CD) in humans [8]; moreover, it has been linked to some extradigestive diseases such as type 1 diabetes (T1D) [9]–[11] and multiple sclerosis (MS) [6], [12]. Indeed, an increased serological prevalence of anti-MAP antibodies (Abs) have been reported in T1D patients from Sardinia [9]–[11], from continental Italy [12], elsewhere [13], and in MS Sardinian subjects [14].

Sera from all the participants included in this case-control study (107 HT patients and 100 HCs) were tested by indirect enzyme-linked immunosorbent assay (ELISA) in order to investigate the prevalence of anti-MAP3865c and anti-ZnT8 antibodies in Sardinia. Abs produced in reaction to MAP3865c peptides are capable of cross-reacting with the homologue ZnT8 peptides of the human body, including the ones located in the thyroid tissue. Indeed, ZnT8 is primarily expressed in the pancreatic β-cell, but it is also specifically expressed in the follicle epithelial cells and parafollicular cells of the thyroid gland [15]. In view of the evidence accounting for a cross-recognition of MAP3865c/ZnT8 homologues sequences in T1D subject [9], [10], [12] and due to the recently proposed theory which pictures MAP as one of the HT environmental trigger, acting trough a molecular mimicry mechanism [4], [5], we decided to investigate the sero-prevalence of anti-MAP/ZnT8 Abs among Sardinian HT patients. To date, this is the first study producing experimental evidence accounting for an association between MAP presence and HT disease.

## Material and Methods

### Subjects

The participants enrolled in this case-control study were 107 individuals affected by Hashimoto's thyroiditis (n = 107; 9 male, 98 female; mean age 45.2±14.6 years), and 100 age and sex-matched healthy controls (HCs). HC subjects were blood donors at the University Hospital of Sassari, with no history of autoimmune disease. All the HT subjects enrolled were attending the Endocrinology Unit of the University Hospital of Sassari, Italy and were diagnosed either according to their history of hypothyroidism with positive antithyroid antibodies, or when there was a diffuse lymphocytic infiltration bilaterally on the pathology report. Patient's details are provided in Table 1. Serum samples were collected from Vacutainer serum tubes.

**Table 1:** Hashimoto's Thyroiditis patients.

*Identity	Gender	Age at incl.	‡Anti TG	† Anti TPO	MAP3865c_125–133_	Znt8_178 –186_	MAP3865c_133–141_	Znt8_186 –194_	MAP3865c_262–275_
HT.1	F	56	NA	NA	−	−	−	−	−
HT.2	F	63	NA	NA	−	−	−	−	−
HT.3	F	73	+	+	−	−	−	−	−
HT.4	F	NA	−	+	−	−	−	−	−
HT.5	F	52	−	NA	+	+	+	+	+
HT.6	F	31	NA	NA	+	−	+	+	+
HT.7	F	43	NA	NA	−	−	−	−	−
HT.8	F	53	+	+	+	+	+	+	−
HT.9	F	51	+	+	−	−	−	−	−
HT.10	F	62	NA	NA	−	−	−	−	−
HT.11	F	20	NA	NA	−	−	−	−	−
HT.12	F	63	+	+	−	−	−	−	+
HT.13	F	36	−	+	−	−	−	−	−
HT.14	F	34	+	+	+	+	+	+	+
HT.15	F	36	−	+	−	−	−	−	+
HT.16	F	55	−	+	−	−	−	−	−
HT.17	F	66	NA	NA	+	+	+	+	+
HT.18	M	45	−	NA	+	+	−	+	+
HT.19	F	63	−	−	−	−	−	−	−
HT.20	F	33	+	−	+	+	−	−	−
HT.21	M	60	NA	NA	+	+	−	−	−
HT.22	F	32	NA	NA	−	−	−	−	−
HT.23	F	18	NA	NA	−	−	−	−	−
HT.24	M	57	−	+	−	−	−	−	−
HT.25	F	22	NA	NA	−	−	−	+	−
HT.26	F	52	+	+	+	+	+	+	+
HT.27	F	56	NA	NA	−	−	−	−	−
HT.28	F	40	NA	NA	−	−	−	−	−
HT.29	F	59	−	−	−	−	−	−	−
HT.30	F	53	−	+	−	−	−	−	−
HT.31	F	26	+	+	−	−	−	−	−
HT.32	F	65	−	−	+	+	+	+	−
HT.33	F	48	+	+	−	−	−	−	−
HT.34	F	66	+	+	−	−	−	−	−
HT.35	F	58	NA	NA	−	−	−	−	−
HT.36	M	44	−	−	−	−	−	−	−
HT.37	F	59	+	+	−	−	−	−	−
HT.38	F	40	+	+	+	+	+	+	+
HT.39	F	35	NA	NA	−	−	−	−	−
HT.40	F	44	+	−	−	−	−	−	−
HT.41	M	14	NA	NA	+	+	−	−	−
HT.42	M	73	NA	NA	−	−	−	−	−
HT.43	F	43	NA	NA	−	−	−	−	−
HT.44	F	62	−	−	−	−	−	−	−
HT.45	M	46	+	+	+	+	−	−	+
HT.46	F	49	+	NA	−	−	−	−	−
HT.47	F	61	+	+	−	−	−	−	−
HT.48	F	51	−	+	+	−	−	+	−
HT.49	F	62	−	−	+	+	+	+	−
HT.50	F	42	NA	NA	−	−	−	−	−
HT.51	F	54	NA	NA	−	−	−	−	−
HT.52	F	53	−	+	−	−	−	−	−
HT.53	F	15	+	+	−	−	−	−	−
HT.54	F	46	NA	NA	−	−	+	+	−
HT.55	F	45	NA	NA	−	−	−	−	−
HT.56	F	51	NA	NA	−	−	−	−	−
HT.57	F	54	NA	NA	−	−	−	−	−
HT.58	F	17	NA	NA	+	+	+	+	−
HT.59	M	48	+	+	−	−	−	−	−
HT.60	F	28	+	+	−	−	−	−	−
HT.61	F	37	NA	NA	−	−	−	−	+
HT.62	F	35	+	+	+	−	−	−	−
HT.63	F	36	−	−	+	+	+	+	+
HT.64	F	14	+	+	−	−	−	−	−
HT.65	F	54	NA	NA	−	−	−	−	−
HT.66	F	44	+	+	−	−	−	−	−
HT.67	F	45	−	−	−	−	−	−	−
HT.68	F	28	NA	NA	+	+	+	−	+
HT.69	F	62	+	+	−	−	−	−	−
HT.70	F	45	−	NA	−	−	−	−	−
HT.71	F	60	NA	NA	−	−	−	−	−
HT.72	F	45	NA	NA	−	−	−	−	−
HT.73	F	45	NA	NA	+	+	+	−	+
HT.74	F	61	+	+	−	−	−	−	−
HT.75	M	58	+	+	−	−	−	−	−
HT.76	F	49	NA	NA	−	−	−	−	−
HT.77	F	33	NA	NA	+	+	+	+	+
HT.78	F	47	−	+	+	+	+	+	+
HT.79	F	53	NA	NA	−	−	+	−	−
HT.80	F	54	NA	NA	+	+	+	+	+
HT.81	F	47	NA	NA	−	−	−	−	−
HT.82	F	41	NA	NA	−	−	−	−	−
HT.83	F	58	NA	NA	−	−	−	−	−
HT.84	F	73	−	+	−	−	−	−	−
HT.85	F	46	NA	NA	−	−	−	−	−
HT.86	F	66	+	−	−	−	−	−	−
HT.87	F	39	NA	NA	−	−	−	−	−
HT.88	F	47	NA	NA	−	−	−	−	−
HT.89	F	40	+	+	+	−	+	−	+
HT.90	F	16	NA	NA	−	−	−	−	−
HT.91	F	63	NA	NA	−	−	−	−	−
HT.92	F	51	NA	NA	−	−	−	−	−
HT.93	F	41	NA	NA	+	+	+	+	−
HT.94	F	40	+	−	−	−	−	−	−
HT.95	F	53	−	−	−	−	−	−	−
HT.96	F	59	NA	NA	−	−	−	−	+
HT.97	F	35	−	−	−	−	−	−	−
HT.98	F	52	NA	NA	−	−	−	−	−
HT.99	F	23	NA	NA	−	−	−	−	−
HT.100	F	43	NA	NA	−	−	−	−	−
HT.101	F	21	NA	NA	−	−	−	−	−
HT.102	F	52	NA	NA	−	−	−	−	−
HT.103	M	12	+	+	+	+	+	+	+
HT.104	F	63	NA	NA	−	−	−	−	+
HT.105	F	10	NA	NA	+	−	−	−	−
HT.106	F	27	−	−	+	+	+	+	+
HT.107	F	60	NA	NA	−	−	−	−	−

*HT: positive Hashimoto's Thyroiditis patient, n = 107; mean age 45.2±14.6 years.

‡Positive if >100 U/ml.

†Positive if >10 U/ml.

NA: Not available.

+and -: Antibody-positive and Antibody-negative patient, respectively.

### Ethical statement

All participants signed an informed consent to be enrolled in our study. The study protocols were approved by the ethics committee of the University Hospital of Sassari, Italy.

### Peptides

Peptides MAP3865c_125–133_ (MIAVALAGL) and MAP3865c_133–141_ (LAANFVVAL) along with their respective homologous peptides ZnT8_178–186_ (MIIVSSCAV), ZnT8_186–194_ (VAANIVLTV) and MAP3865c_262–275_ (DSARVLRDARAVLS) were synthesized at >90% purity (LifeTein, South Plainfield, NJ 07080, USA).

### ELISA

Indirect ELISAs to detect Abs specific for MAP3865c/ZnT8 homologues peptides and anti-MAP3865c_262–275_ were carried out as described elsewhere [9]. Finest cut-off points were identified by Receiver operating characteristic (ROC) curves analyses, setting the specificity at 93% (i.e., Ab+ HCs ≤7% and the corresponding sensitivity was chosen accordingly. Data was normalized to a positive control serum included in all essays run, whose Ab-reactivity was set at 10.000 arbitrary units (AU)/ml.

### Auto-antibodies assays

Anti-TPO and anti-TG antibodies titers were measured by chemiluminescence methodology in the serum of 53 and 51 subjects respectively, using the Liaison Anti-TPO kit (DiaSorin, Italy) for anti-TPO assay with normal values ranging from 0–10 unit/ml and the Liaison Anti-Tg kit (DiaSorin, Italy) for anti-TG assay with normal values ranging from 0–100 units/ml according to the producer's instructions.

### Statistical analysis

Statistical analyses were performed by Graphpad Prism 6.0 software.

## Results

**Figure 1:**
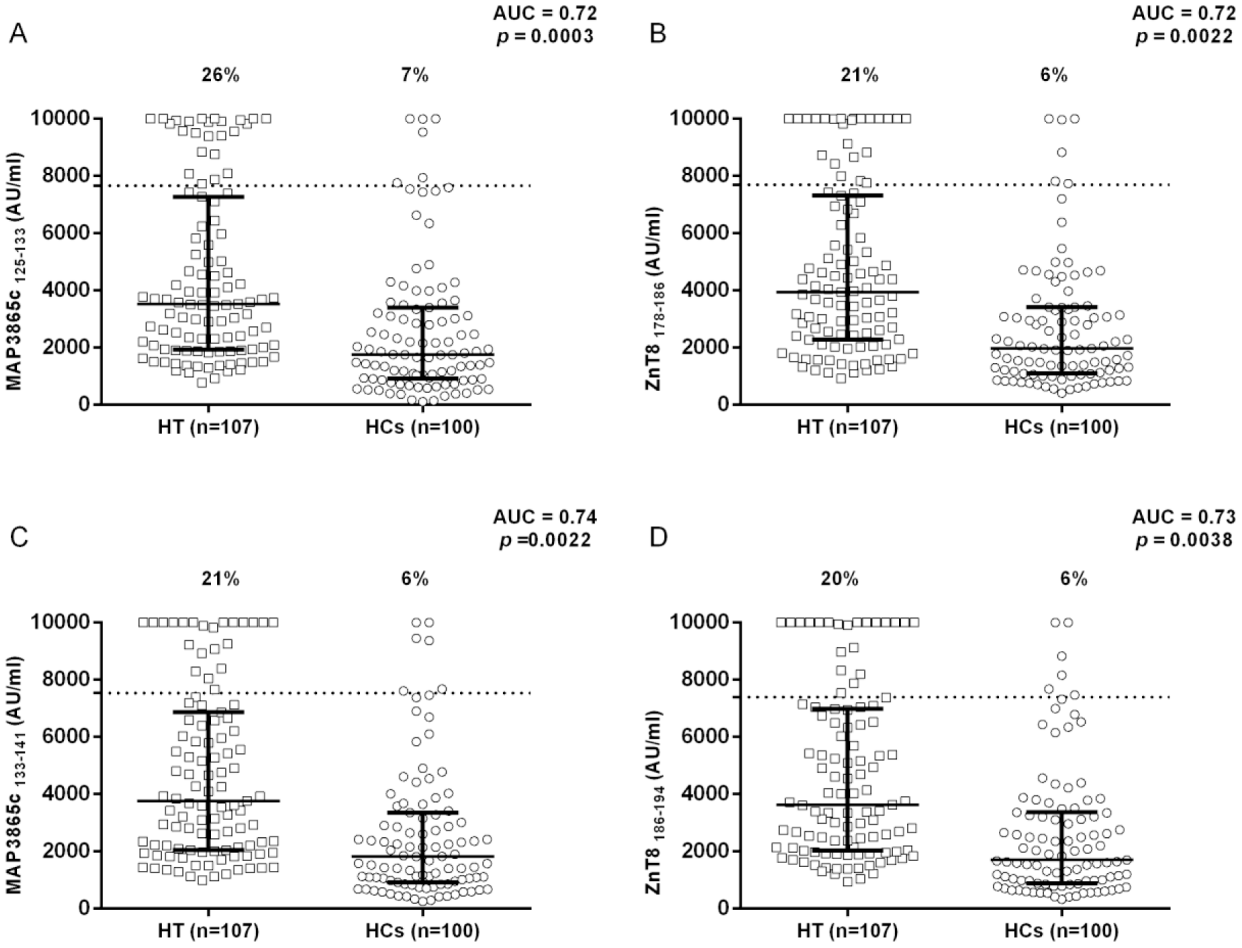
Scatter plot representation of ELISA values in Hashimoto's thyroiditis patients compared to healthy controls. Scatter plots for MAP3865c_125–133_ (**A**) and its homologous ZnT8_178–186_ (**B**) and MAP3865c_133–141_ (**C**) and its homologous ZnT8_186–194_ (**D**) representing the IgG antibodies distribution against these epitopes in Hashimoto's thyroiditis patients and healthy subjects. The number of serum samples in each group is indicated. Dotted lines indicate the cutoff, while bars indicate the corresponding median ± interquartile range. The percent fraction of antibody positive sera is indicated on top of each distribution, whereas AUC and *p* values are given in the top right corner.

**Figure 2:**
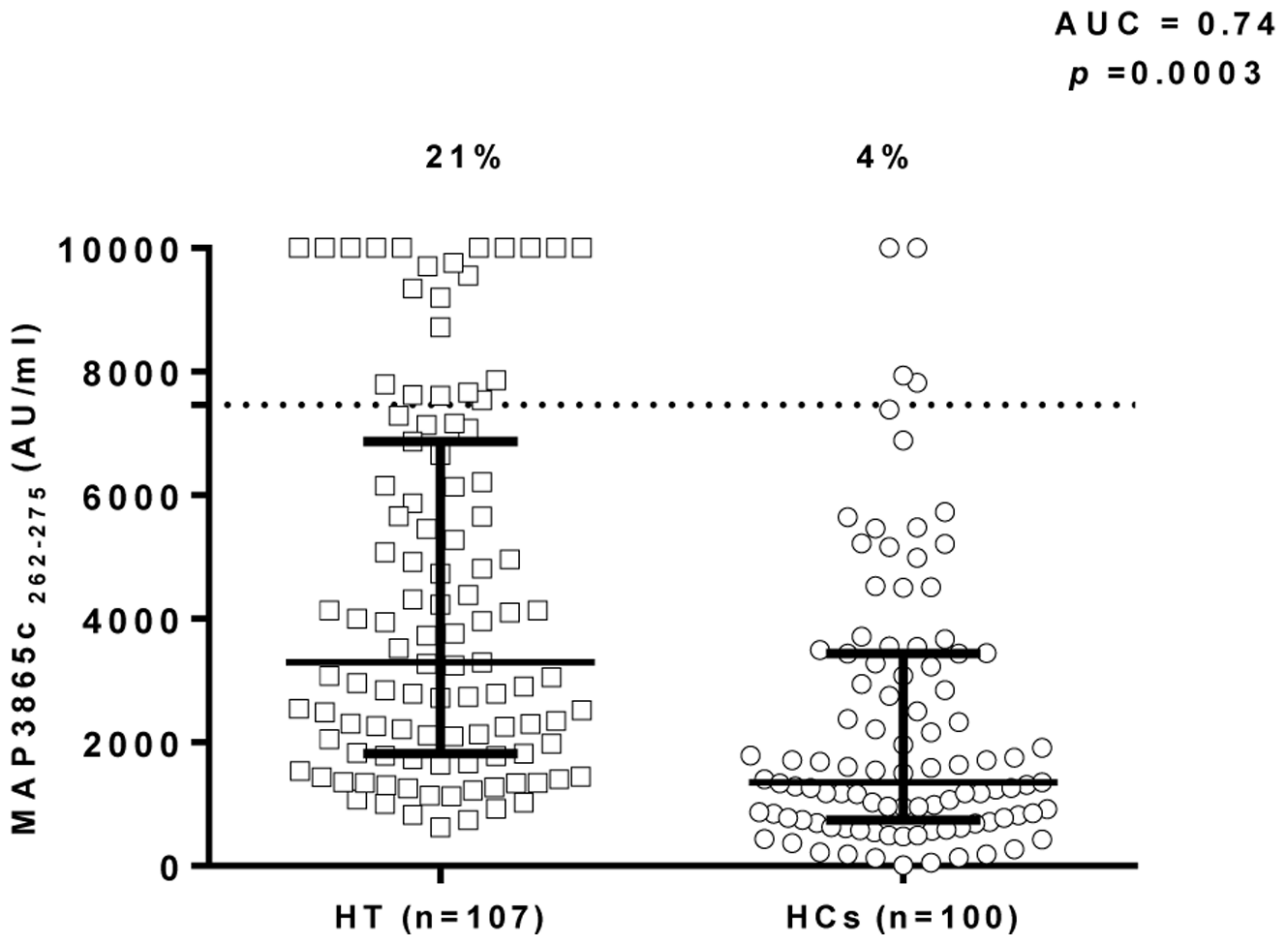
Scatter plots representing the IgG antibodies distribution against MAP3865_262–275_ C-terminal epitope in Hashimoto's thyroiditis patients compared to healthy controls. Data representation is the same as in Figure 1.

We investigated whether MAP3865c/ZnT8 homologues peptides, which were highly immunogenic in T1D subjects, could be recognized by HT Sardinian patients. Five MAP3865c/ZnT8 peptides, four belonging to the fourth trans-membrane domain [9] and one newly designed C-terminal peptide MAP3865c_262–275_ (DSARVLRDARAVLS) were examined in 107 HT, and 100 age matched HCs via our previously set up indirect ELISA [9]. The five peptides were highly recognized proving detectable reactivity. Results are summarized in Fig. 1 and Fig. 2.

MAP 3865c_125–133_ Abs were detected in 26% of HT subjects and in 7% of HCs, this difference was statistically significant (Fisher exact test: *p* = 0.0003; area under ROC curves AUC = 0.72; Fig. 1A).

ZnT8_178–186_ Ab-reactivity was slightly lower than the one showed by its homologue MAP3865c_125133_ when comparing HT with HCs (21% and 6%, respectively; *p* = 0.0022; AUC = 0.72; Fig. 1B).

The homologous MAP3865c_133–141_ and ZnT8_186–194_ peptides (Fig. 1C–D) were recognized by 21% and 20% of HT patients, but only in 6% of HCs (AUC 0.74 and 0.73 respectively; *p* = 0.0022 for MAP3865c_133–141_ and *p* = 0.0038 for ZnT8_186–194_).

Abs against MAP3865c_262–275_ were detected in 21% of HT and in 4% of HC subjects, this difference being statistically significant (Fisher exact test: *p* = 0.0003; AUC = 0.74; Fig. 2).

**Figure 3:**
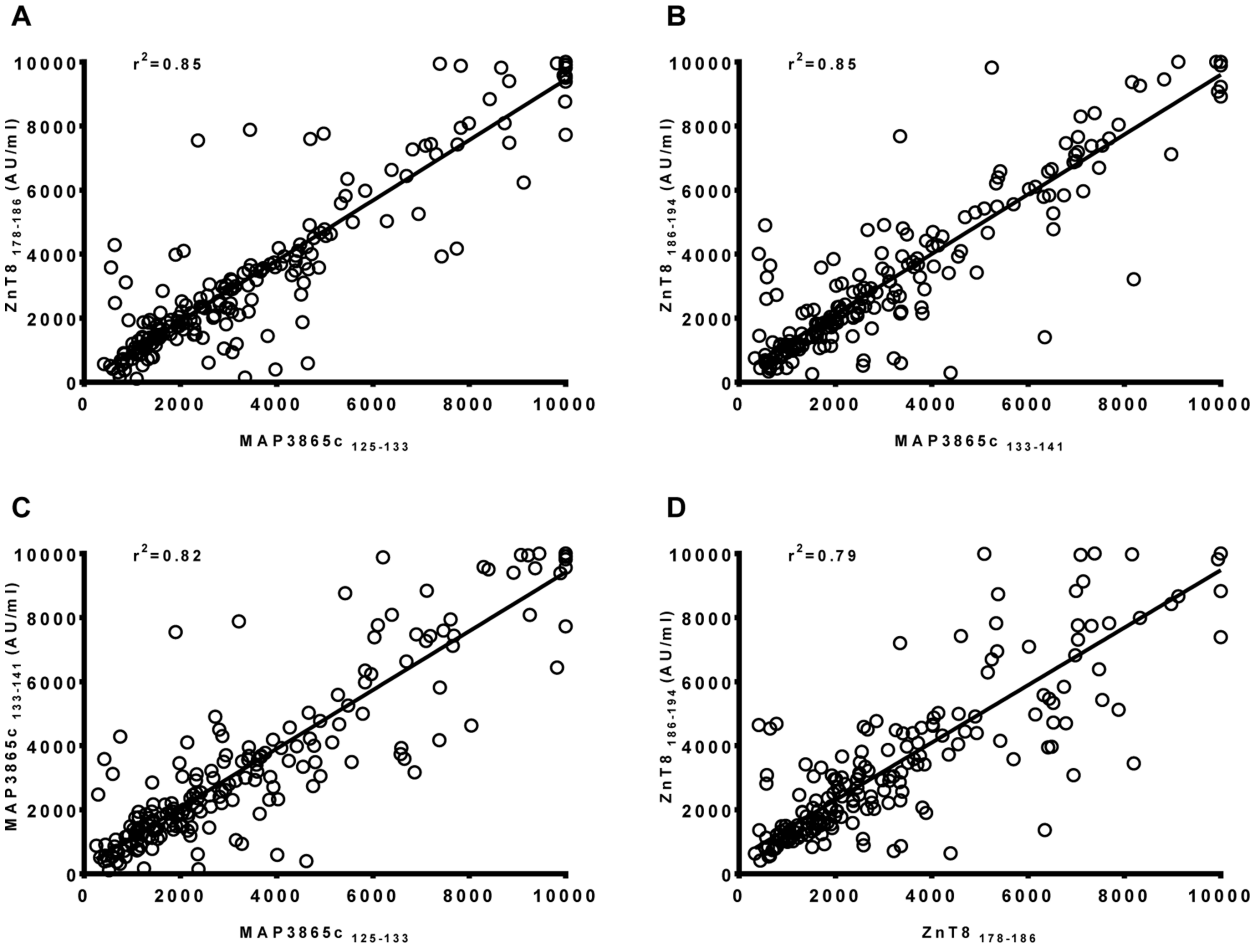
Relationship between titers of MAP3865c and ZnT8-reactive Abs recognizing different epitopes. A significant correlation was found between titers of IgG antibodies recognizing MAP3865c_125–133_ and its homologous ZnT8_178–186_ epitope (**A**) and MAP3865c_133–141_ and its homologous ZnT8_186–194_ (**B**). A significant correlation was also observed between MAP3865c_125–133_ and its consecutive MAP3865c_133–141_ epitope (**C**) and ZnT8_178–186_ and its consecutive ZnT8_186–194_ (**D**).

A correlation analysis revealed that there was a high degree of correlation between titers of Abs recognizing MAP3865c and ZnT8 homologous sequences in both HT patients and HCs (Fig. 3). R^2^ = 0.85 for both MAP3865c_125–133_ vs ZnT8_178–186_ and MAP3865c_133–141_ vs ZnT8_186–194_ (Fig. 3 A–B). A similar correlation was found for MAP3865c_125–133_ vs MAP3865c_133–141_ and ZnT8_178–186_ ZnT8_186–194_ (Fig. 3C–D; r^2^ = 0.82 and 0.74, respectively).

## Discussion

MAP asymptomatic infection and sero-reactivity are highly prevalent amongst T1D and MS Sardinian subjects. The aforementioned diseases are acknowledged to be among the most prevalent autoimmune disease in Sardinia (fourth and fifth, respectively). Indeed, Sardu *et al*. reported that HT is the most common autoimmune disease in Sardinia, and that individuals affected by one autoimmune disease are more likely to develop a second autoimmune disorder [16]. All of this pointing out that there might be a common pathogenic mechanism behind these autoimmune diseases. Besides, MAP has been called forth as a potential environmental trigger of HT [4], [5]. The link between MAP and HT was postulated after establishing the presence of viable MAP organism in few Italian subjects suffering from HT disease. MAP was detected by reverse transcription-PCR [4], [5].

The objective of this study was to verify the robustness of the postulated association investigating whether ZnT8/MAP peptides could be cross- recognized in HT individuals. We asked whether Abs against these epitopes may be at play in HT in Sardinian subjects. This hypothesis pictures MAP as a new HT environmental trigger, capable of imitating immunogenic components of the thyroid gland, acting mainly through a molecular mimicry mechanism. Interestingly, ZnT8 is expressed in pancreatic β-cells and in the follicle epithelial cells and parafollicular cells lining the thyroid follicle.

Our study shows that Abs against mycobacterial (MAP3865c_125–133_, MAP3865c_133–141_ MAP3865c_281–287_) in conjunction with Abs against human (ZnT8_178–186_ and ZnT8_186–194_) peptides are highly common in HT from Sardinia, but barely detectable in age and sex matched HCs.

Of note, titers against MAP3865c_133–141_ and MAP3865c_125–133_ epitopes were very similar to the one displayed by the human homologous peptides (ZnT8_186–194_, and ZnT8_178–186_) in HT and healthy subjects, with a percent fraction of Ab+ sera ranging from 20% to 26%.

Indirect ELISAs highlighted a very similar reactivity against MAP3865c/Znt8 homologues epitopes accounting for an Ab-mediated cross-recognition. Indeed, correlation analyses confirm that Abs recognizing homologous MAP3865c/ZnT8 sequences segregate within the same sera.

The fact that ZnT8 has not been found expressed by medullary thymic epithelial cells [17], may suggest that negative selection of ZnT8-reactive T cells may be ineffective.

Therefore, it will be interesting to investigate the existence of autoreactive effector T-cells capable of recognizing MAP3865c/ZnT8 homologous sequences. To date, our newly designed C-terminal peptide MAP3865c_262–275_ (DSARVLRDARAVLS) present a 53% amino acid identity with ZnT8_326–340_ (DSQVVRREIAKALSK) epitope, which was recently reported to be a CD4 (+) T cell target in T1D patients [18]. Noteworthy, this is the first report which significantly associates MAP infection with HT. It is still early to conclude whether this is due to a loss of tolerance toward these epitopes, in fact we here provided only evidence accounting for an association between Abs positivity for ZnT8 and MAP homologue epitopes in Sardinian HT individuals. Further studies are clearly needed to test the hypothesis that MAP may represent an important environmental factor capable of triggering HT. Likewise, genetic polymorphism of the host and other environmental factors need to be explored.
